# Supplementary material for: Seroprevalence and correlates of SARS-CoV-2 neutralizing antibodies from a population-based study in Bonn, Germany
Source: Nat Commun. 2021 Apr 9;12:2117. doi: 10.1038/s41467-021-22351-5 (PMC8035181; doi:10.1038/s41467-021-22351-5)
Supplement: Supplementary file 1 — Supplementary Information [file 41467_2021_22351_MOESM1_ESM.pdf]

Supplementary Information

Seroprevalence and correlates of SARS-CoV-2 neutralizing antibodies from a population-based study in Bonn, Germany

Authors

N. Ahmad Aziz[1,2], Victor M. Corman[3,4], Antje K.C. Echterhoff[1], Marcel A. Müller[3,4], Anja Richter[3]; Antonio Schmandke[1], Marie Luisa Schmidt[3,4], Thomas H. Schmidt[1], Folgerdiena M. de Vries[1], Christian Drosten[3,4], Monique M.B. Breteler[1,5*]

[1] *Population Health Sciences, German Center for Neurodegenerative diseases (DZNE), Bonn, Germany.*

[2] *Department of Neurology, Faculty of Medicine, University of Bonn, Bonn, Germany*

[3] *Institute of Virology, Charité-Universitätsmedizin Berlin, corporate member of Freie Universität Berlin, Humboldt-Universität zu Berlin, and Berlin Institute of Health, Berlin, Germany*

[4] *German Center for Infection Research (DZIF), Berlin, Germany*

[5] *Institute for Medical Biometry, Informatics and Epidemiology (IMBIE), Faculty of Medicine, University of Bonn, Germany*

[*] **Corresponding author:**

Prof. Dr. Monique M.B. Breteler, MD, PhD

Population Health Sciences

German Center for Neurodegenerative diseases (DZNE) within the Helmholtz Association

Venusberg-Campus 1, Building 99

53127 Bonn, Germany

Tel: +49-228-43302-929

E-mail: monique.breteler@dzne.de

Supplementary Table 1. Sample characteristics (*Group II*) stratified by serostatus

Serostatus	N	Age (y)	Sex (f)	Number of comorbidities	Number of symptoms
ELISA-	350	55.1 (12.5)	0.61	1.0 (1.3)	5.2 (4.6)
ELISA±/IFT-/PRNT-	3	52.3 (11.0)	0.33	2.7 (2.3)	3.3 (3.5)
ELISA+/IFT+/PRNT-	2	68.0 (17.0)	0.50	0.5 (0.7)	6.5 (2.1)
ELISA+/IFT+/PRNT+	4	55.2 (4.6)	0.75	1.0 (1.2)	8.0 (6.2)
ELISA+/IFT±/PRNT+	1	58	0	1	3

Legend: Values represent mean (standard deviation) for continuous variables and fractions for categorical variables. Abbreviations: f = female, IFT = immunofluorescence test, PRNT = plaque reduction neutralization test, y = years.

Supplementary Table 2. Titers of neutralizing antibodies at baseline and follow-up in participants with neutralizing antibodies at baseline.

	$PRNT_{50}$		$PRNT_{90}$	
Subject	Baseline	Follow-up	Baseline	Follow-up
1	1:80	<1:20	<1:20	<1:20
2	1:40	<1:20	<1:20	<1:20
3	>1:80	1:20	1:20	<1:20
4*	>1:80	>1:80	1:20	>1:80
5	1:40	1:40	<1:20	<1:20
6	1:40	1:20	1:20	<1:20
7*	>1:80	>1:80	1:20	1:40
8	1:20	<1:20	<1:20	<1:20
9	>1:80	>1:80	1:20	1:20
10	1:40	1:20	<1:20	<1:20
11*	1:40	1:40	<1:20	1:20
12	1:40	1:20	<1:20	<1:20
13	>1:80	1:40	1:40	<1:20
14*	>1:80	>1:80	1:20	1:40
15	>1:80	>1:80	1:20	1:20
16	1:40	1:20	<1:20	<1:20
17	1:20	<1:20	<1:20	<1:20
18	>1:80	>1:80	1:80	1:40
19	1:40	1:20	<1:20	<1:20
20	>1:80	>1:80	1:40	1:40
21	1:40	1:20	<1:20	<1:20
22*	>1:80	>1:80	1:20	1:80

Legend: Neutralizing antibody titers were measured as the concentration of serum to reduce the number of plaques by either 50 or 90% (defined as $PRNT_{50}$ or $PRNT_{90}$, respectively) and categorized as <1:20 (i.e. undetectable), 1:20, 1:40, 1:80 or >1:80. Asterisks (*) mark individuals in whom the levels of neutralizing antibodies increased: Four of these five individuals with a clear rise in $PRNT_{90}$ levels were already in the highest $PRNT_{50}$ category. Only one individual with a borderline increase of $PRNT_{90}$ levels (i.e. from the <1:20 to the 1:20 category) had $PRNT_{50}$ titers of 1:40 at both visits; this latter case is likely due to inter-assay variability in the semi-quantitative assessment of neutralizing antibody titers. Abbreviations: PRNT = plaque reduction neutralization test.

Supplementary Table 3. Validation of SARS-CoV-2 serological assays using N=100 pre-pandemic human sera from the same cohort (i.e. Group I).

N sera	EUROIMMUN anti-SARS-CoV-2 IgG ELISA*	In-house recombinant spike-based immunofluorescence test**	Plaque reduction neutralization test ($PRNT_{50}$ and $PRNT_{90}$)
100	0/100	0/100	0/100

Legend:
* Cut-off IgG ratio ≥1.1. All samples were below this cut-off threshold, with one out of 100 sera, with an IgG ratio of 0.96, rated as borderline (see **Supplementary Fig. 5**, image ID5312).
Three out of 100 sera had high background signals at 1:10 dilution, but could still be rated as negative (see **Supplementary Fig. 5, images ID5306, ID5312, ID5297).

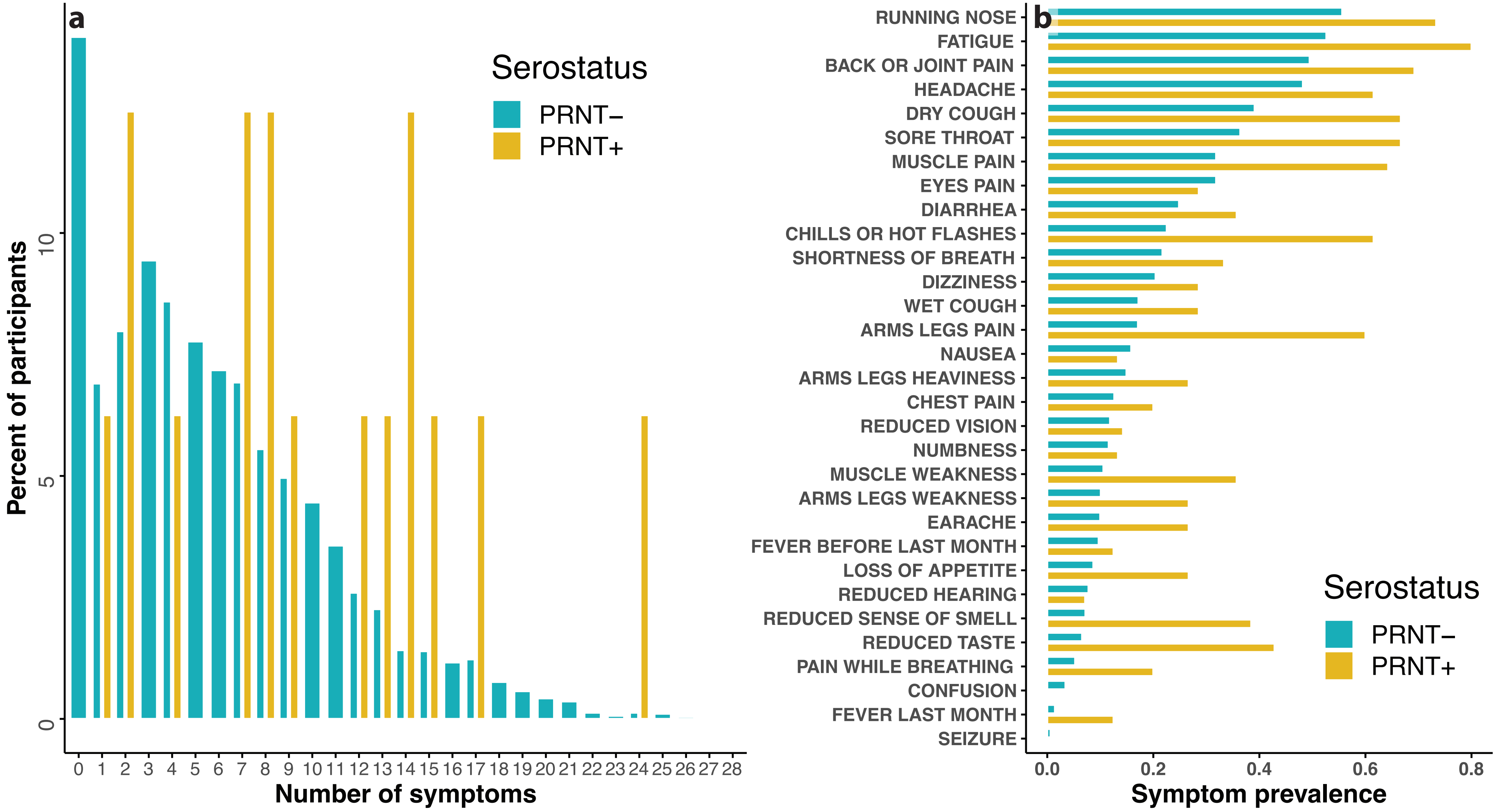

Supplementary Fig. 1 Proportion of participants reporting symptoms, stratified by serostatus and number of symptoms (**a**) or type of symptoms (**b**). Abbreviations: PRNT = plaque reduction neutralization test.

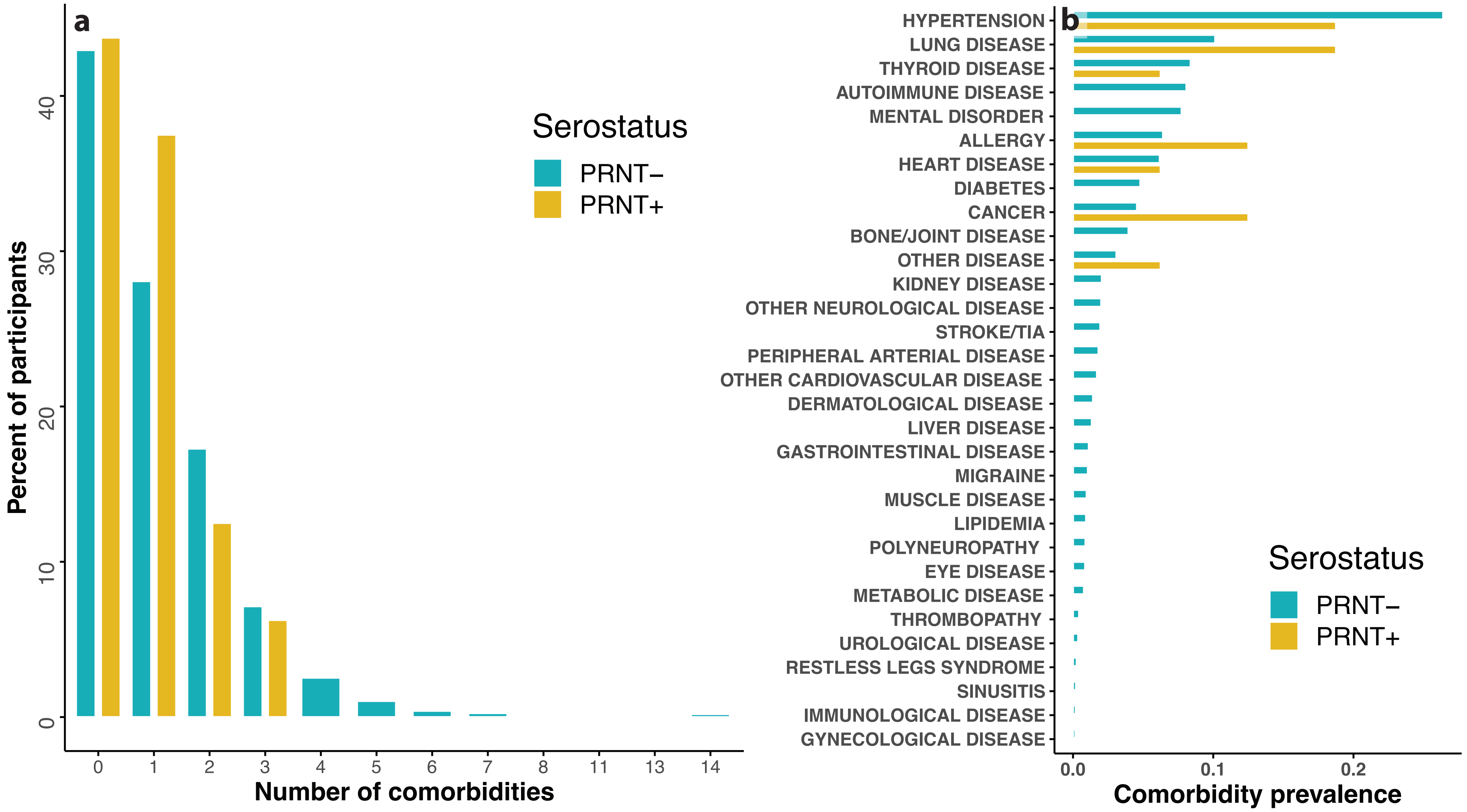

Supplementary Fig. 2 Proportion of participants reporting comorbidities, stratified by serostatus and number of comorbidities (**a**) or type of comorbidity (**b**). Abbreviations: PRNT = plaque reduction neutralization test.

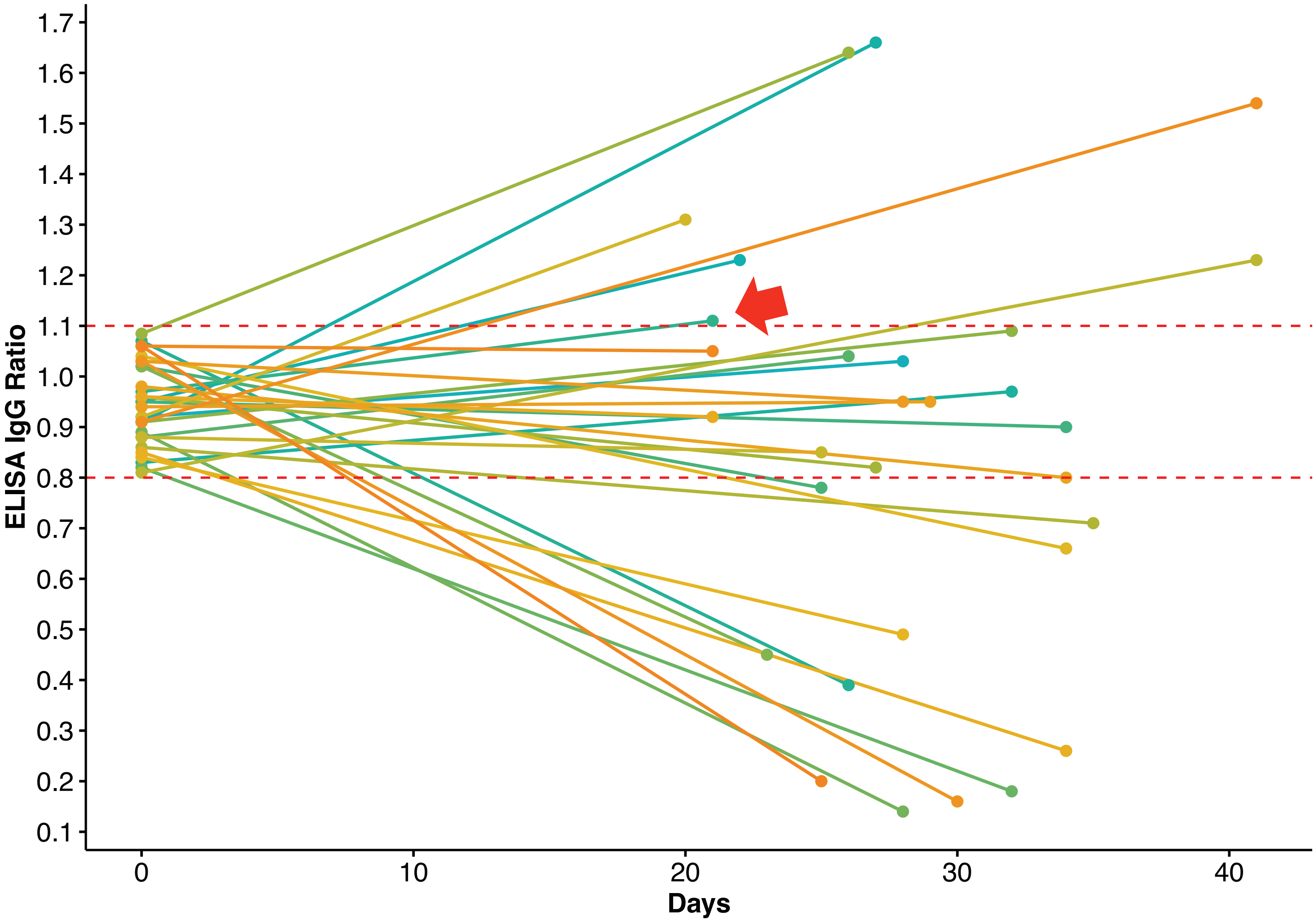

Supplementary Fig. 3 All individuals with an IgG ratio in the indeterminate range (i.e. between 0.8 and 1.1) at baseline were reassessed after at least 20 days. The arrow marks the individual who had detectable neutralizing antibodies at baseline, but not at the follow-up visit. Neutralizing antibodies were not detectable in any participant at follow-up. The horizontal dotted lines represent the borders of the indeterminate range.

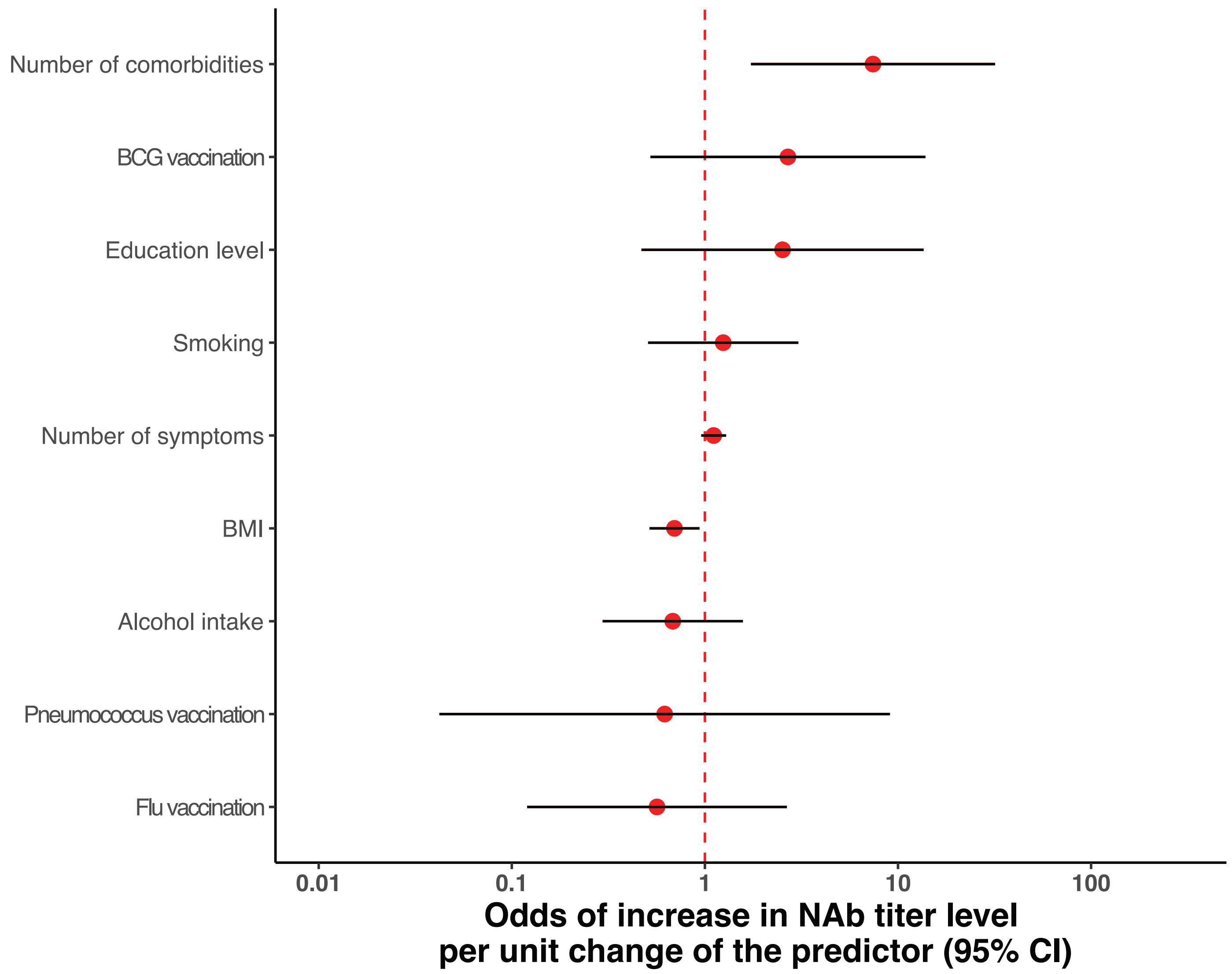

Supplementary Fig. 4 Titers of neutralizing antibodies (as assessed by $PRNT_{50}$) were positively associated with having more co-morbidities, and inversely related to a higher body mass index (BMI). All estimates are based on generalized estimating equations using a cumulative logit link function to account for the ordinal nature of the response variable and intra-individual clustering of the repeated measurements, and adjusted for follow-up time, age and sex. Sample size: 22 individuals with SARS-CoV-2 neutralizing antibodies at baseline. The red dots represent the odds ratio point estimates, while the whiskers depict the corresponding 95% CIs, on a logarithmic scale. Abbreviations: CI = confidence interval, NAb = neutralizing antibody, PRNT = plaque reduction neutralization test.

Supplementary Figure 5

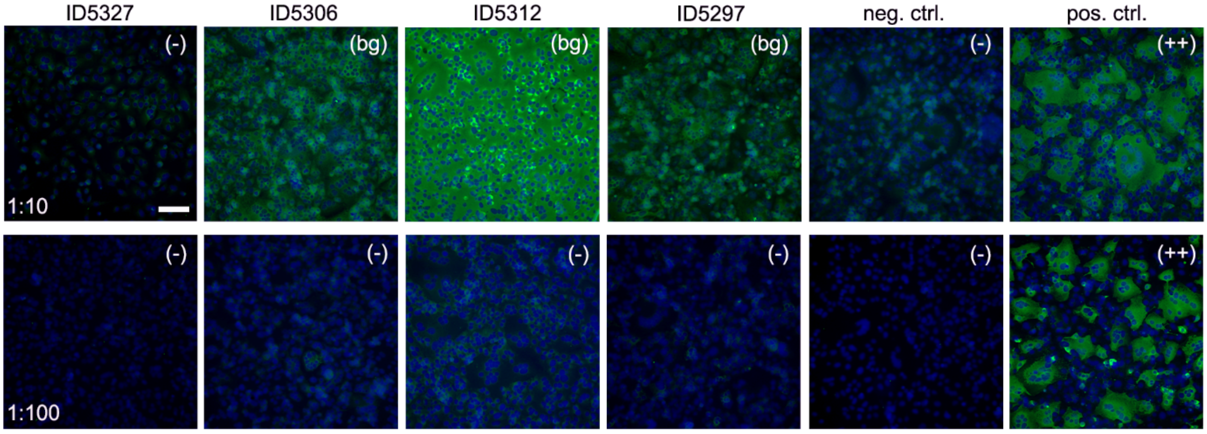

Supplementary Fig. 5 Validation of the in-house anti-SARS-CoV-2 antibody recombinant immunofluorescence test (rIFT). VeroFM cells were transfected with recombinant SARS-CoV-2 spike-carrying plasmids and fixed with ice-cold acetone/methanol (1:1) 24 hours post transfection. Human sera were applied in 1:10 and 1:100 dilutions using EUROIMMUN sample buffer, and slides were incubated for 1 hour at 37°C. Secondary detection was done using a goat anti-human immunoglobulin Alexa488 (1:200). Slides were mounted in prolonged mounting medium containing DAPI (Thermo Scientific). Samples with ID5327 to ID5297 examples from N=100 pre-pandemic sera all tested by rIFT. ID5327 represents the typical staining pattern of an ELISA-negative serum. ID5306-5297 represent samples with non-specific background signals at 1:10 dilutions including ID5312, which showed borderline reactivity in the ELISA (OD ratio=0.96). Each sample was measured once. Images were generated using a Leica Thunder3D microscope. All images were taken using the same microscopic settings and were processed in parallel in Adobe Photoshop. Scale bar = 100 µm. Ratings indicate either neg. (-), background (bg) or positive (+ to +++) immunofluorescence signals. A study-unrelated pre-pandemic negative serum was used as negative control (neg. ctrl). As positive control (pos. ctrl.) a confirmed COVID-19 patient serum was applied.
