# Supplementary material for: Seroprevalence and correlates of SARS-CoV-2 neutralizing antibodies from a population-based study in Bonn, Germany
Source: Nat Commun. 2021 Apr 9;12:2117. doi: 10.1038/s41467-021-22351-5 (PMC8035181; doi:10.1038/s41467-021-22351-5)
Supplement: Supplementary file 3 — Reporting Summary [file 41467_2021_22351_MOESM3_ESM.pdf]

nature research

Corresponding author(s): Monique M.B. Breteler

Last updated by author(s): Mar 3, 2021

Reporting Summary

Nature Research wishes to improve the reproducibility of the work that we publish. This form provides structure for consistency and transparency in reporting. For further information on Nature Research policies, see our Editorial Policies and the Editorial Policy Checklist.

Statistics

For all statistical analyses, confirm that the following items are present in the figure legend, table legend, main text, or Methods section.

n/a	Confirmed	
☐	☒	The exact sample size (*n*) for each experimental group/condition, given as a discrete number and unit of measurement
☐	☒	A statement on whether measurements were taken from distinct samples or whether the same sample was measured repeatedly
☐	☒	The statistical test(s) used AND whether they are one- or two-sided<br>*Only common tests should be described solely by name; describe more complex techniques in the Methods section.*
☐	☒	A description of all covariates tested
☐	☒	A description of any assumptions or corrections, such as tests of normality and adjustment for multiple comparisons
☐	☒	A full description of the statistical parameters including central tendency (e.g. means) or other basic estimates (e.g. regression coefficient) AND variation (e.g. standard deviation) or associated estimates of uncertainty (e.g. confidence intervals)
☐	☒	For null hypothesis testing, the test statistic (e.g. *F*, *t*, *r*) with confidence intervals, effect sizes, degrees of freedom and *P* value noted<br>*Give P values as exact values whenever suitable.*
☒	☐	For Bayesian analysis, information on the choice of priors and Markov chain Monte Carlo settings
☐	☒	For hierarchical and complex designs, identification of the appropriate level for tests and full reporting of outcomes
☐	☒	Estimates of effect sizes (e.g. Cohen's *d*, Pearson's *r*), indicating how they were calculated

Our web collection on statistics for biologists contains articles on many of the points above.

Software and code

Policy information about availability of computer code

Data collection	Participants received a paper questionnaire that they were asked to complete at home and return to the study centre. The data from the questionnaires was then manually entered and uploaded to the study database. No specific software was used for data collection. Adobe Illustrator was used for formatting of all figures, and additionally, Adobe Photoshop was used for formatting of Supplementary Figure 5.
Data analysis	All analyses were performed in R (base version 3.6.1). The following R packages were used for the statistical analyses: geepack (version 1.3-1), multgee (version 1.7.0) and stats (version 3.6.3).

For manuscripts utilizing custom algorithms or software that are central to the research but not yet described in published literature, software must be made available to editors and reviewers. We strongly encourage code deposition in a community repository (e.g. GitHub). See the Nature Research guidelines for submitting code & software for further information.

Data

Policy information about availability of data

All manuscripts must include a data availability statement. This statement should provide the following information, where applicable:

- Accession codes, unique identifiers, or web links for publicly available datasets
- A list of figures that have associated raw data
- A description of any restrictions on data availability

The Rhineland Study's dataset is not publicly available because of data protection regulations. Access to data can be provided to scientists in accordance with the Rhineland Study's Data Use and Access Policy. Requests for further information or to access the Rhineland Study's dataset should be directed to RS-DUAC@dzne.de.

Field-specific reporting

Please select the one below that is the best fit for your research. If you are not sure, read the appropriate sections before making your selection.

- [x] Life sciences
- [ ] Behavioural & social sciences
- [ ] Ecological, evolutionary & environmental sciences

For a reference copy of the document with all sections, see nature.com/documents/nr-reporting-summary-flat.pdf

Life sciences study design

All studies must disclose on these points even when the disclosure is negative.

Sample size	Due to implementation of this serosurvey in an ongoing cohort study, the study group was already predefined and therefore no a priori sample size calculation was performed. This serosurvey was conducted in two groups. Group I consisted of all living participants who had been enrolled in the Rhineland Study until March 18th, 2020 (N=5427). Their participation in the study was therefore unrelated to attitude to or experience with SARS-CoV-2. Group II consisted of individuals who were eligible for but had not yet participated in the Rhineland Study. They actively approached us to indicate their willingness to participate in the serosurvey (N=597), which we allowed for those who agreed to become prospective participants in the Rhineland Study. Participation in this group was thus motivated by the prospect of being tested: we used this group to estimate the effect of selection bias in SARS-CoV-2 serosurveys.
Data exclusions	This serosurvey was based on the Rhineland Study, an ongoing community-based cohort study in Bonn, Germany. All inhabitants aged 30 years and above of two geographically defined areas are invited to participate in the Rhineland Study. The sole exclusion criterion for participation in the Rhineland Study is insufficient command of the German language to provide informed consent. For the present study we included data on all individuals who participated in the serosuvey.
Replication	This serosurvey was an observational cross-sectional study concerning the seroprevalence and correlates of neutralizing antibodies against SARS-CoV-2 in Bonn, Germany, in the period between April 24th and June 30th, 2020. Therefore, there were no experimental findings that would be amenable to replication.
Randomization	Given the observational design of the study, randomization procedures were not applicable.
Blinding	Given the observational design of the study, blinding procedures were not applicable.

Reporting for specific materials, systems and methods

We require information from authors about some types of materials, experimental systems and methods used in many studies. Here, indicate whether each material, system or method listed is relevant to your study. If you are not sure if a list item applies to your research, read the appropriate section before selecting a response.

Materials & experimental systems

n/a	Involved in the study	
[ ]	[x]	Antibodies
[x]	[ ]	Eukaryotic cell lines
[x]	[ ]	Palaeontology and archaeology
[x]	[ ]	Animals and other organisms
[ ]	[x]	Human research participants
[x]	[ ]	Clinical data
[x]	[ ]	Dual use research of concern

Methods

n/a	Involved in the study	
[x]	[ ]	ChIP-seq
[x]	[ ]	Flow cytometry
[x]	[ ]	MRI-based neuroimaging

Antibodies

Antibodies used	The levels of immunoglobulin G (IgG) antibodies against SARS-CoV-2 were measured using a commercially available ELISA (EUROIMMUN, Lübeck, Germany). According to the manufacture's product sheet, applying a cut-off of >1.1 for defining seropositivity results in an estimated sensitivity of 94.4% (at >10 days of infection) and specificity of 99.6%. Jackson, no. 109-545-088, Alexa Fluor488-conjugated AffiniPure Goat anti-human IgG (H+L) (Lot 147499) in a 1:200 dilution was used for the immunoassay validation experiments (Supplementary Fig. 3 and Supplementary Figure 5).
Validation	Our independent in-house validation experiments of this assay using serum samples from 119 plasma donors after convalescence from mild to moderate COVID-19 (as documented by positive swab nucleic acid testing), as well as 110 healthy subjects (with either no history of COVID-19-typical symptoms and no risk contacts, or negative pharyngeal swab SARS-CoV-2 nucleic acid testing), were in line with the manufacturer's reported values yielding a sensitivity of 86.8% and a specificity of 100%. We performed two additional confirmatory tests in all those individuals whose ELISA assay results were either positive (i.e. >1.1) or borderline (i.e. between 0.8 and 1.1). Confirmatory tests consisted of an in-house recombinant immunofluorescence test and a plaque reduction neutralisation test (PRNT) to specifically check for the presence of neutralizing antibodies against SARS-CoV-2.

Human research participants

Policy information about studies involving human research participants

Population characteristics	Population characteristics (including age, sex and education level) are summarized in Table 1 of the article.
Recruitment	This serosurvey was conducted in two groups. Group I consisted of all living participants who had been enrolled in the Rhineland Study until March 18th, 2020 (N=5427). Their participation in the study was therefore unrelated to attitude to or experience with SARS-CoV-2. Group II consisted of individuals who were eligible for but had not yet participated in the Rhineland Study. They actively approached us to indicate their willingness to participate in the serosurvey (N=597), which we allowed for those who agreed to become prospective participants in the Rhineland Study. Participation in this group was thus motivated by the prospect of being tested. We provide seroprevalence estimates for each group (i.e. Group I and II) separately: given the high response rate in Group I (i.e. 88%) the risk of selection bias in Group I, i.e. our main target population, is very low. On the other hand, the risk of selection bias in Group II is high: we specifically included this group by design to estimate the magnitude of self-selection bias in SARS-CoV-2 seroprevalence studies.
Ethics oversight	Approval to undertake the Rhineland Study was obtained from the ethics committee of the University of Bonn, Medical Faculty. The Rhineland Study is carried out in accordance with the recommendations of the International Conference on Harmonization (ICH) Good Clinical Practice (GCP) standards (ICH-GCP) after obtainment of written informed consent from all participants in accordance with the Declaration of Helsinki. No separate ethical approval for this serosurvey was required given its embedding in the Rhineland Study, the ethical mandate of which already covered follow-up measurements, including collection of serial bio-samples.

Note that full information on the approval of the study protocol must also be provided in the manuscript.
